# Maximum Cumulative Ratio (MCR) as a Tool for Assessing the Value of Performing a Cumulative Risk Assessment

**DOI:** 10.3390/ijerph8062212

**Published:** 2011-06-16

**Authors:** Paul S. Price, Xianglu Han

**Affiliations:** Toxicology and Environmental Research and Consulting, The Dow Chemical Company, 1803 Building, Midland, MI 48674, USA; E-Mail: xhan2@Dow.com

**Keywords:** cumulative, risk, exposure, mixtures

## Abstract

Due to the vast number of possible combinations of chemicals to which individuals are exposed and the resource-intensive nature of cumulative risk assessments, there is a need to determine when cumulative assessments are most required. This paper proposes the use of the maximum cumulative ratio (MCR) as a tool for this evaluation. MCR is the ratio of the cumulative toxicity received by an individual from exposure to multiple chemical stressors to the largest toxicity from a single chemical stressor. The MCR is a quantitative measure of the difference in an individual’s toxicity estimated using a chemical-by-chemical approach and using an additive model of toxicity. As such, it provides a conservative estimate of the degree to which individuals’ toxicities could be underestimated by not performing a cumulative risk assessment. In an example application, MCR is shown to be applicable to the evaluation of cumulative exposures involving up to 81 compounds and to provide key insights into the cumulative effects posed by exposures to multiple chemicals. In this example, MCR values suggest that individuals exposed to combinations of chemicals with the largest Hazard Indices were dominated by the contributions of one or two compounds.

## 1. Introduction

### 1.1. The Concern for Cumulative Toxicity from Concurrent Exposure to Multiple Chemicals

Humans are constantly exposed to multiple chemicals from multiple sources [1–4]. However, regulatory programs such as TSCA in the United States and REACH in the European Union evaluate risks on a chemical-by-chemical basis and do not require the consideration of cumulative exposures when determining human health effects. It has been asserted that the determination of toxicity on this basis could underestimate the total toxicity to individuals [4]. The chemical-by-chemical approach is believed to underestimate toxicity when the combined exposures to chemicals result in a cumulative toxicity that exceeds the toxicity of the most toxic of the individual chemicals. In these instances, a chemical-by-chemical approach could find that each chemical posed no unacceptable risk, but the mixture of chemicals could result in unacceptable effects.

Tools for evaluating risk from cumulative exposures have been developed by the U.S. Environmental Protection Agency [3], and other organizations [5–7]. Tiered approaches for evaluation of cumulative exposures have also been developed by the World Health Organization (WHO) [6,7]. However, there has been relatively little investigation into the magnitude of the toxicity missed if a cumulative risk assessment is not performed. This paper addresses this gap and is intended to be fully compatible with the WHO framework.

### 1.2. The Maximum Cumulative Ratio

In this paper we describe a simple tool, the Maximum Cumulative Ratio (MCR) that provides a quantitative measure of the magnitude of the toxicity that is underestimated by not performing a cumulative risk assessment. The MCR is defined as the ratio of the toxicity received by an individual from exposures to multiple chemicals (cumulative toxicity) to the largest toxicity received by the individual from any one chemical (maximum chemical toxicity).

This paper applies the concept of the MCR to estimates of toxicity derived from dose additive models. Dose additive models include simple conservative screening approaches that do not consider mechanism of action or the target organs (WHO Tier 1 assessments) and more refined assessments that do consider these factors (WHO Tier 2 assessments). Under additive models, a risk ratio is created by dividing the dose of an individual chemical by a measure of the chemical’s toxicity. In the case of the Hazard Index approach [4], this measure is the “permitted” dose for the chemical. “Permitted” doses include regulatory standards and guidance values such as the Reference Dose, Population Adjusted Dose, Allowable Daily Dose, Tolerated Daily Intake, or Derived No Effect Level. Other additive models of toxicity include the Toxicity Unit approach used in aquatic toxicology [3,8].

The values of MCR for individuals in a population (MCR_i_) determined using the Hazard Index (HI) approach is calculated using the following equations. The measure of cumulative toxicity received by the i^th^ individual in a population exposed to *n* chemicals is given by the individual’s hazard index (HI_i_):

(1) $${\text{HI}}_{\text{i}}=\sum^{\text{n}}{\text{HQ}}_{\text{ij}}$$

where HQ_ji_ is the hazard quotient contributed from the dose of the j^th^ of the *n* chemicals to the i^th^ individual (D_ij_). The value of HQ_ji_ is given by:

(2) $${\text{HQ}}_{\text{ij}}=\frac{{\text{D}}_{\text{ij}}}{{\text{PD}}_{\text{j}}}$$

PD_j_ is the permitted dose of the j^th^ chemical for humans. The maximum of the chemical-specific toxicities for the i^th^ individual is given by:

(3) $${\text{MHQ}}_{\text{i}}=\text{Max}({\text{HQ}}_{\text{i}})$$

The value of MCR for the i^th^ individual (MCR_i_,) is given by:

(4) $${\text{MCR}}_{\text{i}}=\frac{{\text{HI}}_{\text{i}}}{{\text{MHQ}}_{\text{i}}}$$

Recognition of the importance of the ratio of the cumulative toxicity to the maximum toxicity from any one chemical in assessing the toxicity of mixtures is not new. The ratio has been used in the field of aquatic toxicology in the evaluation of the effects of mixtures. In 1981, Konemann [8] proposed the use of this ratio as part of a quantitative strategy for the determination of the type of joint action of mixtures of chemicals for fish. Konemann noted that the range of the ratio is bounded by 1 and n, where *n* is the number of chemicals in a mixture. The ratio has a value of 1 for mixtures where all of the mixture’s toxicity comes from one component. A mixture will have a value of *n* when all the chemicals are present in equitoxic doses.

Junghans *et al.* [9] observed that the ratio could be used to predict when dose additive and independent action models of a mixture’s toxicity produce similar or divergent estimates of toxicity. When ratio values for a mixture are close to 1, the dose additive and independent action models produce virtually identical results. The authors go on to note that the toxicities of mixtures with ratios close to 1 are dominated by the contributions of a few components.

**Figure 1.**
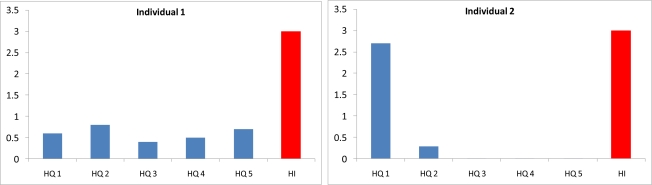
Illustration of MCR as a predictor of the need for cumulative risk assessments.

The concept of MCR builds on Junghans *et al.*’s observation that the ratio is a measure of whether individuals’ cumulative exposures are dominated by a single chemical or are the result of the contribution of many chemicals [9]. As the illustration in Figure 1 demonstrates, dominance of one chemical is a critical factor in determining the need for a cumulative risk assessment. In this illustration, two individuals are assumed to have cumulative exposures to five chemicals. The hazard indices of the two individuals are 3. For the first individual the values of the Hazard Quotients for the five chemicals are, 0.6, 0.8, 0.4, 0.5, and 0.7. For the second individual the values are 2.7, 0.29, 0.008, 0.001, and 0.001 (Figure 1). For the first individual no single chemical is a concern (all Hazard Quotients are less than 1.0), yet the cumulative measure of toxicity is 3 times the level of concern. Thus, a cumulative risk assessment is necessary for individual 1. For the second individual, a chemical-by-chemical based approach reaches the same conclusion as a cumulative risk assessment—the exposures of the second individual are unacceptable. Thus there is less value in performing a cumulative risk assessment in the second case.

The values of MCR for the two individuals are different. The value of *MCR* for the first individual is 3.8. The value of *MCR* for the second is 1.1. This suggests that values of MCR that are close to 1 indicate a lower need for a cumulative risk assessment. This property of the MCR indicates that the measure can be used to rank the relative importance of performing cumulative risk assessments for different groups of chemicals and different exposed populations.

In addition, MCR can be used as a quantitative estimate of the toxicity missed a cumulative risk assessment is not performed. The estimate of maximum hazard to an individual that can be identified under a chemical-by-chemical approach is MHQ_i_. The estimate of the maximum toxicity identified under a cumulative risk assessment (assuming additivity) is HI_i_. MCR is the ratio of HI_i_ to MHQ_i_ and therefore the fraction of the toxicity that is missed in the i^th^ individual by not performing a cumulative risk assessment is:

(5) $${\text{Missed}\;\text{toxicity}}_{\text{i}}=1-\frac{1}{{\text{MCR}}_{\text{i}}}$$

For example, an MCR value of 2 indicates that 50% of an individual’s hazard index would be missed if a chemical-by-chemical method is used to assess the individual instead of a cumulative risk assessment. For a mixture with an MCR value of 1.25, the missing portion is 20%.

### 1.3. Application of the Maximum Cumulative Ratio to Mixtures of Pest Protection Products in Surface Waters of the U.S.

In this example, MCR values are determined for cumulative exposures to multiple pest protection products (PPPs) and degradation products of PPPs measured in surface water samples collected under the National Water-Quality Assessment (NAWQA) program [10]. The cumulative risk analysis performed on the mixtures follows the Tier 1 approach described in the WHO guidance for the assessment of mixtures [7]. In a Tier 1 assessment, the effects of all components are assumed to be addressed by an additive model of toxicity. (Under WHO guidance, mechanism of action is evaluated in Tier 2 assessments.)

Exposures are characterized using a generic exposure scenario that is based on conservative exposure assumptions. The scenario assumes that the levels of chemicals observed in the samples occur in drinking water supplies. The doses of each chemical in the mixture are estimated by assuming that the water is consumed at a rate of 2 liters per day by an adult who weighs 60 kilograms. The permitted doses of the chemicals, PD_j_, are based on chronic non-cancer standards for the chemicals. Although the measured data are only a “snapshot” of levels of chemicals at one point in time, the levels of the chemicals are assumed to be constant over time, thus allowing the use of the chronic standards. Note: The long-term average levels at the sampling locations would result in a smaller range of concentrations of chemicals. (Day-to-day variation would be averaged out.) In particular, the upper bound values will be lower for the long-term averages than for the grab samples. Thus use of chronic standards for the values of PD_j_ will tend to overestimate the values of HQ_i_ for samples with high concentrations of PPPs.

The values of MCR and HI from an individual’s exposure to the chemicals in each of the samples are determined. These data are used to investigate the relationships between MCR, HI, and the number of chemicals in each mixture (*n*). The questions that are investigated are:

- Do MCR values vary across the samples, and if so, what is the range of MCR values?
- Are the values of MCR closer to *n* or 1?
- Are MCR values correlated with *n*?
- Are MCR values correlated with the values of HI_i_ for the individuals exposed to the chemicals in the samples?
- How does the presence of chemicals that occur at levels below the detection limits affect the determination of the values of HI and MCR associated with exposure to a mixture?

The relationships between HI and MCR are investigated since the MCR values that are of most interest to regulators are those that come from the mixtures of higher toxicity. The relationship between MCR and *n* is investigated since it could provide insights to the values of MCR for complex mixtures where *n* is very large. The impact of non-detects is investigated since non-detects are a significant source of uncertainty in cumulative risk assessments [11].

## 2. Experimental Section

### 2.1. Materials

The NAQWA is a program operated by the U.S. Geological Survey and is the first U.S. survey of PPPs and their degradation products performed on a national scale in the U.S. [10]. The NAWQA dataset was chosen for several reasons. First, it is a publically available dataset that includes a large number of samples from a wide range of locations. Second each sample was analyzed for a large number of chemicals. Finally, permitted doses for chronic exposures, PD_j_, are available for virtually all of the chemicals analyzed for in the samples.

Data on chemical levels in samples of surface water collected under the NAQWA survey are available from the U.S. Geological Survey’s internet site. The dataset can be downloaded at [12]. These data used here were collected over the first decade of the monitoring program (4,380 samples from 1992–2001) and reflect agricultural practices of that period. The number of analytes measured in each of the samples varies by date and location and range from 12 to 81. The number of chemicals detected in the samples ranged from 0 to 29. In total, 83 chemicals were analyzed in one or more samples.

Table 1 presents values of PD_j_ for 81 of the 83 chemicals. The values of PD_j_ are largely composed of chronic Reference Doses and chronic Population Adjusted Doses established by the Office of Pesticide Programs of the U.S. Environmental Protection Agency (Table 2). These criteria are based on multiple endpoints and target tissues; however as discussed above, in a Tier 1 assessment the effects are assumed to be additive. Permitted doses were not identified for two chemicals (Fenuron and Neburon). Because of the absence of PD values for these chemicals, they were not included in the cumulative assessment. Omitting these chemicals could result in lower estimates of cumulative risk; however, the frequencies of detection of the compounds in the samples are low (0.2% and 0.1% respectively). Because the compounds rarely occur, omitting the compounds is unlikely to change the general findings for the cumulative exposures to the mixtures.

### 2.2. Preliminary Analyses of Survey Data and Development of a Subset of Mixture Samples

The NAWQA dataset includes samples with no detectable levels of chemicals or with only one or two detections. The goal of the assessment is to investigate cumulative risks for individuals exposed to a number of chemicals. In order to obtain a dataset where exposure to a significant number of chemicals occurs, water samples with detectable levels of less than five chemicals were removed from the dataset.

In the NAWQA samples, there are a large numbers of analytes with levels below the detection limits (non-detects). This presents a challenge for characterizing cumulative exposures using monitoring data. While, assessors should not assume that non-detected compounds are absent from samples [11], in large numbers non-detects can drive the estimates of the toxicity of the mixture and the values of MCR. In order to investigate the impact of non-detects on HI and MCR values, the data was analyzed using two assumptions, Case 1 where non-detects are set to zero, and Case 2 where non-detects are assumed to have concentrations equal to the detection limit (DL) divided by the square root of two (DL/2^0.5^). Better methods for estimating the impacts of non-detects are available; however, since the method used here does not have a significant impact on results of samples of greater human health concern (see the “Results and Discussion” Section) this approach is deemed to be sufficient. The assumption of chemical being present at the DL/2^0.5^ is a method frequently used for estimating non-detects [11].

### 2.3. Statistical Analyses

Trends in the relationships between HI, MCR, and *n* were evaluated by plotting the data in Microsoft Excel spreadsheet and performing statistical analyses in JMP 8.0.2 (SAS Institute Inc.). Nonparametric correlations between HI, MCR, and *n* were performed using Kendall’s rank correlation test. Correlations were evaluated using data on values of individual samples and the medians values for samples that are grouped based on *n*. Medians were not determined for groups with less than 10 samples. Wilcoxon Test (a nonparametric test) was used to compare the MCR values of samples of HI values less than 1 with samples of HI values greater than 1.

## 3. Results and Discussion

### 3.1. Results

The final dataset consists of 3,099 samples. Data on the compositions of the mixtures are given in Table 3. As would be expected in a survey analyzing for large numbers of chemicals, there are more non-detects than detects in the samples.

Table 4 presents the values of MCR and HI for Cases 1 and 2. Values of HI ranged over seven orders of magnitude. The inclusion of the contributions of the non-detects had a significant impact on the minimum and mean values of the MCR and HI but not on the maximum values of HI. The number of samples with HI values greater than 1 was similar for the two cases, 63 for Case 1 and 66 for Case 2. Table 5 give the means and ranges of MCR value for samples with HI values above and below 1. MCR values in samples with HI values less than 1 were two fold higher when non-detects were considered but largely unchanged for the samples with HI values greater than 1. These findings suggest that in this dataset non-detects are not an important factor in the determination of values of HI and MCR for samples with values of HI greater than 1.

**Figure 2.**
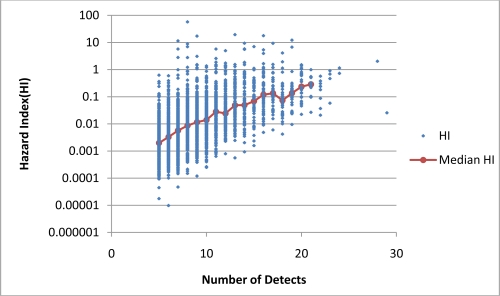
The relationship between HI and *n* for Case 1. HI and *n* have a strong positive correlation, Kendall’s tau-b value of 0.44 (p < 0.0001) based on all samples and Kendall tau-b value of 0.96 (p < 0.0001) for medians of grouped samples.

**Figure 3.**
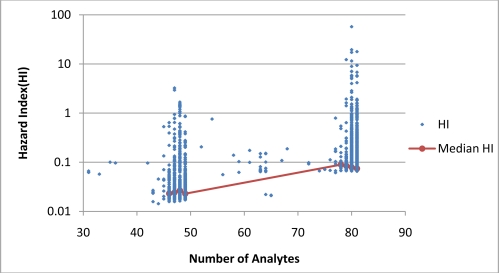
The relationship between HI and *n* for Case 2. HI and *n* have a weakly positive correlation, Kendall’s tau-b value of 0.34 (p < 0.0001) based on all samples. Correlation based on medians of grouped samples is not statistically significant (p > 0.05).

Figures 2 and 3 present plots of the range of HI values in samples grouped based on *n*. The distributions of samples by *n* are very different in the two cases. In Case 1 the value of *n* is equal to the number of detected chemicals while in Case 2 *n* is equal to the number of analytes. In the NAQWA dataset samples were taken over a 10 year period using a variety of analytical methods; however, most samples were analyzed using variation on one of two analytical methodologies. These two methodologies tested for 48 and 80 analytes [10]. As a result the numbers of analytes in the samples tend to cluster around values of 48 and 80. In contrast the number of detections is more evenly distributed.

For Case 1, HI was found to be positively correlated with *n* based on analyses of both sample values and the medians for the grouped samples (p < 0.0001). The median values of HI were 100-fold larger in samples with 20 detected chemicals than in samples with five detected compounds. For Case 2, HI was weakly correlated with *n* based on sample values and was not statistically correlated with *n* based on median HI values of the grouped samples. These analyses suggest that when *n* is based on the number of detected chemicals (Case 1) it is a strong predictor of the value of HI. When *n* is based on the number of analytes (Case 2) the correlation is much weaker.

**Figure 4.**
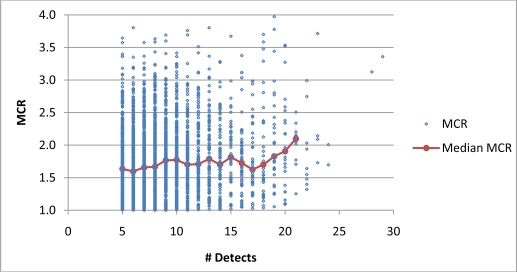
The relationship between MCR and *n* for Case 1. MCR and *n* have a weakly positive correlation, Kendall’s tau-b value of 0.08 (p < 0.0001) based on all samples and Kendall tau-b value of 0.55 (p < 0.01) for medians of grouped samples.

**Figure 5.**
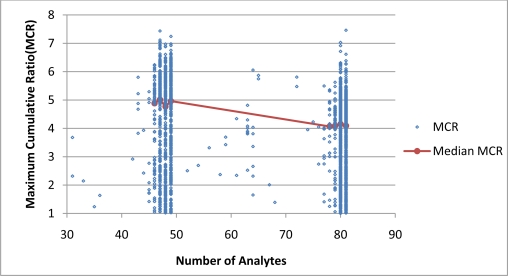
The relationship between MCR and *n* for Case 2. MCR and *n* have a weakly negative correlation, Kendall’s tau-b value of −0.12 (p < 0.0001) based on all samples. Correlation based on medians of grouped samples is not statistically significant (p > 0.05).

Figures 4 and 5 present plots of the range of MCR values as a function of *n* for Cases 1 and 2. When based on data of all individual samples, the correlations between MCR and *n* are weakly positive for Case 1 and weekly negative for Case 2. When based on median MCR values of groups, MCR was found to be weakly correlated with the number of detects (Figure 4) but not the number of analytes (Figure 5). These findings indicate that in this dataset, *n* was not a strong predictor of MCR values in either Case 1 or 2.

**Figure 6.**
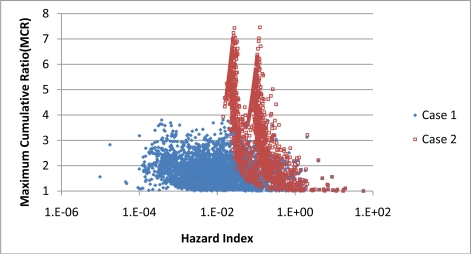
Plot of MCR and HI values for all samples. HI and MCR are negatively correlated, Kendall’s tau-b values of −0.16 (p < 0.001) for Case 1 and values of −0.18 (p < 0.0001) for Case 2.

**Figure 7.**
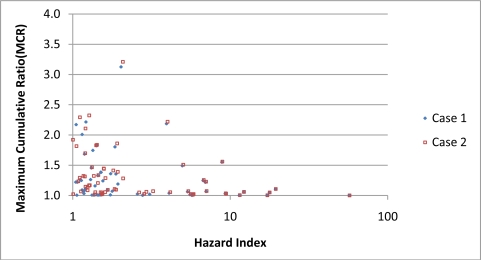
Plot of MCR and HI values for samples with HI greater than 1. HI and MCR are negatively correlated, Kendall’s tau-b values of −0.22 (p < 0.001) for Case 1 and values of −0.30 (p < 0.0001) for Case 2.

Figure 6 presents a plot of MCR *versus* HI for Cases 1 and 2 for all the samples in the final dataset while Figure 7 presents samples with values of HI larger than 1. In Figure 6, the relationship between MCR and HI appears very different for Case 1 and 2. In Case 1, the plot of the MCR samples appear as a diffuse cloud that tapers off at higher value of HI. In contrast, MCR values in Case 2 rise sharply as HI value decreases and separate into two distinct peaks. Additional analysis of the data indicated that the data points in the two peaks came from samples analyzed using the two different analytical methodologies. The left peak came from samples analyzed using the methodology that detected 48 chemicals and the right peak from the methodology that detected 80 samples. As Figure 7 indicates, the impact of the non-detects on the MCR and HI is minimal for samples that have HI values greater than 1.

Results from the Wilcoxon Test showed that the MCR values of samples with HI greater than 1 are significantly lower than samples with HI values less than 1. These differences occurred for both Case 1 and 2 (see Table 2). This suggests that MCR values are inversely correlated for higher values of HI. Figures 6 and 7 provide additional evidence for this relationship. Tests of the correlation found statistically significant negative correlations between MCR and HI for the entire dataset and for samples with HI values greater than 1. These finding occurred for both Case 1 and 2. For the samples with HI values greater than 1, the MCR values average 1.26 for Case 1 and 1.31 for Case 2. These values of MCR imply that on average 20–25% of cumulative toxicity predicted using the Tier 1 screening models is missed by not performing a cumulative risk assessment on the mixtures in the samples.

### 3.2. Discussion

The application of the MCR to the cumulative assessment of the risks from chemicals measured in the NAQWA Dataset demonstrates the potential value of the MCR for characterizing the need for cumulative risk assessments. MCR values were determined for the cumulative toxicities of the chemical mixtures in the 3,099 surface water samples. The values of *n* in the NAQWA samples ranged from 5 to 81. This demonstrates that the MCR approach can be applied to large numbers of cumulative exposures and can be applied to relatively complex mixtures when toxicity data are available for the mixture’s components. Non-detects are a significant issue in the analysis of the NAQWA data. Including contributions from non-detects had a significant impact on the estimates of the values of HI and MCR. However, this impact was limited to those samples predicted to have low cumulative toxicity (HI values less than 1).

The values of HI for the samples ranged over five orders of magnitude. The vast majority of the samples (98%) had HI values less than one. Values of MCR range from 1.0–4.0 (mean of 1.8) in Case 1 and 1.0–7.5 (mean of 4.0) in Case 2. These values are much lower than the values of *n* for the samples (31–81 analytes). This indicates that the toxicities of all of the mixtures are dominated by a very small fraction of the compounds present. The values of HI were correlated with *n*, indicating that samples with more detected compounds in general had higher estimates of cumulative toxicity. In contrast, MCR values had little or no increase with *n*. This suggests that in this dataset, higher numbers of chemicals in a mixture do not necessarily indicate an increased need for a cumulative risk assessment. By plotting MCR *versus* HI the analysis demonstrated that many samples have a toxicity that may be seven-fold greater than the toxicity of any one chemical component. However, for the mixtures with higher toxicity (HI values greater than 1) the difference was less than three-fold for all of the samples and averaged only 1.3 fold. The finding of a negative correlation of MCR with HI suggests that the toxicities of the mixtures of the greatest concern are driven by smaller numbers of compounds than the mixtures with minimal toxicity. This implies that the higher toxicity in these samples did not occur as a result of the contribution of multiple chemicals summing to unacceptable levels of toxicity, but rather from the presence of one (or two) chemicals that either were highly toxic or occurred at high concentrations. The final decision on the need to perform a cumulative risk assessment will be determined by many factors; however, in this case performing a Tier 1 cumulative risk assessment would result in only modest changes in the predictions of risk for the more toxic mixtures.

This assessment has focused on cumulative exposures to PPPs and degradation products of PPPs that result from the co-occurrence of the chemicals in surface water samples. The approach can be applied to cumulative exposures that occur from exposures to mixtures of chemicals in soil, air, or on indoor surfaces. The approach can also be applied to cumulative exposures to chemicals from multiple sources when the doses of the chemicals can be defined for a single individual. While not discussed, the MCR approach could be extended to consider non chemical stressors when the impact of those stressors on the toxicity of the chemicals is defined. Finally, as discussed above, the purpose of this analysis is the illustration of the application of the MCR to a real world dataset. The purpose is not to reach any conclusion on the safety of current levels of PPPs in U.S. surface waters. The levels observed in the NAQWA dataset are measures of conditions one to two decades ago and do not necessarily reflect current practices in the U.S. In addition, the exposure assumptions used in this analysis will lead to significant overestimates of actual chronic exposures the mixtures of chemicals in the samples. Many of the samples are taken from surface waters that are not appropriate for drinking water supplies (small streams) and the impacts of water treatment processes on the levels of PPPs are not considered. Under a WHO tier 1 assessment, value of HI greater than 1 indicate that the samples would pass on to Tier 2 and Tier 3 assessments where more realistic exposure and toxicity assumptions would be used [7].

## 4. Conclusions

MCR may provide a useful tool to assess the value of performing a cumulative risk assessment. The approach can be extended to cumulative exposures involving large numbers of chemicals and can be applied to large monitoring datasets. The approach can be applied as part of Tier 1 assessments that use simple additive models of toxicity. The findings provide a quantitative estimate of individuals’ toxicities missed by not performing a cumulative risk assessment. As a result, MCR values could be used as part of a decision process to determine when, and where, future cumulative risk assessments are most needed to protect human health.

## Figures and Tables

**Table 1.** Chronic toxicity standards for chemicals measured in surface water samples.

**Chemical**	**Permitted Dose mg/kg/day**	**Source code^a^**	**Chemical**	**Permitted Dose mg/kg/day**	**Source code**	**Chemical**	**Permitted Dose mg/kg/day**	**Source code**

2,4,5-T	0.01	1	Cyanazine	0.00026	5	Molinate	0.001	3
2,4,5-TP	0.008	1	Dacthal	0.01	2	Napropamide	0.12	2
2,4-D	0.005	2	Dacthal monoacid	0.01	2	Norflurazon	0.015	2
2,4-DB	0.03	2	Diethyl atrazine	0.0018	2^b^	Oryzalin	0.12	2
2,6 Diethylaniline	0.006	3^b^	Diazinon	0.0002	2	Oxamyl	0.001	2
3-Hydroxycarbofuran	0.00006	2^b^	Dicamba	0.45	2	*p,p’*-DDE	0.0005	3
Acetochlor	0.02	3	Dichlobenil	0.015	2	Parathion	0.006	7
Acifluorfen	0.004	2	Dichlorprop	0.036	2	Parathion-methyl	0.00002	2
Alachlor	0.01	2	Dieldrin	0.00005	6	Pebulate	0.0007	2
Aldicarb	0.00027	3	Dinoseb	0.001	1	Pendimethalin	0.1	2
Aldicarb sulfone	0.00027	3	Dinitro-*o*-cresol	0.004	6	Phorate	0.00017	2
Aldicarb sulfoxide	0.00027	3	Disulfoton	0.00013	2	Picloram	0.2	2
alpha-HCH	0.008	3	Diuron	0.003	2	Prometon	0.05	2
Atrazine	0.0019	2	EPTC	0.0025	2	Pronamide	0.027	2
Azinphos-methyl	0.00149	2	Ethalfluralin	0.04	2	Propachlor	0.054	2
Benfluralin	0.005	2	Ethoprop	0.0001	2	Propanil	0.009	2
Bentazon	0.03	2	Fluometuron	0.005	2	Propargite	0.04	2
Bromacil	0.1	2	Fonofos	0.002	2	Propham	0.02	1
Bromoxynil	0.015	2	γ-HCH	0.0003	1	Propoxur	0.005	2
Butylate	0.05	2	Linuron	0.0077	2	Simazine	0.0018	2
Carbaryl	0.01	2	Malathion	0.07	2	Tebuthiuron	0.07	2
Carbofuran	0.00006	2	MCPA	0.0044	2	Terbacil	0.013	2
Chloramben methyl ester	0.014	4	MCPB	0.015	2	Terbufos	0.00005	2
Chlorothalonil	0.02	2	Methiocarb	0.005	2	Thiobencarb	0.01	2
Chlorpyrifos	0.00003	2	Methomyl	0.008	2	Triallate	0.025	2
*cis*-Permethrin	0.25	2	Metolachlor	0.1	2	Triclopyr	0.05	2
Clopyralid	0.15	3	Metribuzin	0.013	2	Trifluralin	0.024	2

a See Table 2;

b PPP metabolites are assumed to have equal toxicity to the parent compound on a molar basis.

**Table 2.** Sources of Toxicity Data Cited in Table 1.

**Source Code from Table 1**	**Source of toxicity data (PD_j_)**

1	USEPA Integrated Risk Information System. http://cfpub.epa.gov/ncea/iris/index.cfm?fuseaction=iris.showSubstanceList.
2	USEPA Office of Pesticide Programs Pesticide Reregistration Status. http://www.epa.gov/opp00001/reregistration/status.htm
3	Regulations.gov. http://www.regulations.gov/#!home
4	http://www.consumersunion.org/pdf/fqpa/ReportCard_appendix1.pdf
5	Minnesota Department of Health. Health Risk Limits for Groundwater 2008 Rule Revision Health Risk Assessment Unit, Environmental Health Division. http://www.health.state.mn.us/divs/eh/risk/guidance/gw/cyanazine.pdf
6	Agency for Toxic Substances and Disease Registry. Toxicological Profiles http://www.atsdr.cdc.gov/ToxProfiles/tp1.pdf
7	USEPA Drinking Water Standards and Health Advisories Table. http://www.epa.gov/region9/water/drinking/files/DWSHATv09.pdf

**Table 3.** The number of chemicals (*n*) in the final set of samples.

	**Minimum Number**	**Maximum Number**	**Average Number**

**Detected Chemicals**	5	29	9
**Non-Detects**	20	76	61
**Number of Chemicals Analyzed for in a Sample**	31	81	70

**Table 4.** Values of HI and MCR for samples in the final dataset.

	**HI Values for Samples**			**MCR Values for Samples**		

	Minimum	Maximum	Average	Minimum	Maximum	Average

Case 1						
Non-Detects = 0	0.00001	57	0.14	1.0002	4.0	1.8
Case 2						
Non-Detects = DL/2^0.5^	0.014	57	0.19	1.001	7.5	4.0

**Table 5.** Comparison of MCR values of samples with HI greater or less than 1.

**HI cutoff**	**HI < 1**			**HI > 1**		

Statistics	Minimum	Maximum	Average	Minimum	Maximum	Average

Case 1	1.002	4.0	1.8	1.0002	3.1	1.3
Case 2	1.024	7.5	4.1	1.0014	3.2	1.3
